# Are preoperative histology and MRI useful for classification of endometrial cancer risk?

**DOI:** 10.1186/s12885-016-2554-0

**Published:** 2016-07-19

**Authors:** Noemie Body, Vincent Lavoué, Olivier De Kerdaniel, Fabrice Foucher, Sébastien Henno, Aurélie Cauchois, Bruno Laviolle, Marc Leblanc, Jean Levêque

**Affiliations:** Gynaecology Department, Rennes University Hospital, Hôpital Sud, Rennes, France; Pathology Department, CHU Pontchaillou, Rennes University Hospital, Rennes, France; Clinical Pharmacology Department, Rennes University Hospital, CIC Inserm 0203, Hôpital Pontchaillou, Rennes, France; Gynaecology Department, Bretagne Atlantique Hospital, Vannes, France; Oncogenesis, Stress and Signaling, ER 4440, CRCL CRLCC Eugène Marquis, Rennes, France; University of Rennes 1, Faculty of Medicine, Rennes, France

**Keywords:** Endometrial cancer, MRI, Endometrial sample, Risk group, Lymphadenectomy

## Abstract

**Background:**

The 2010 guidelines of the French National Cancer Institute (INCa) classify patients with endometrial cancer into three risk groups for lymph node invasion and recurrence on the basis of MRI and histological analysis of an endometrial specimen obtained preoperatively. The classification guides therapeutic choices, which may include pelvic and/or para-aortic lymphadenectomy. The purpose of this study was to evaluate the diagnostic performance of preoperative assessment to help identify intermediate- or high-risk patients requiring lymphadenectomy.

**Methods:**

The study included all patients who underwent surgery for endometrial cancer between January 2010 and December 2013 at either Rennes University Hospital or Vannes Regional Hospital. The criteria for eligibility included a preoperative assessment with MRI and histological examination of an endometrial sample. A histological comparison was made between the preoperative and surgical specimens.

**Results:**

Among the 91 patients who underwent a full preoperative assessment, the diagnosis of intermediate- or high-risk endometrial cancer was established by MRI and histology with a sensitivity of 70 %, specificity of 82 %, positive predictive value (PPV) of 87 %, negative predictive value (NPV) of 61 %, positive likelihood ratio (LR+) of 3.8 and negative likelihood ratio (LR-) of 0.3. The risk group was underestimated in 32 % of patients and overestimated in 7 % of patients. MRI underestimated endometrial cancer stage in 20 % of cases, while endometrial sampling underestimated the histological type in 4 % of cases and the grade in 9 % of cases.

**Conclusion:**

The preoperative assessment overestimated or underestimated the risk of recurrence in nearly 40 % of cases, with errors in lesion type, grade or stage. Erroneous preoperative risk assessment leads to suboptimal initial surgical management of patients with endometrial cancer.

## Background

The FIGO (International Federation of Gynecology and Obstetrics) classification of endometrial cancer and its surgical management was reviewed in 2009–2010 [1]. The preoperative assessment recommended by the French National Cancer Institute (INCa) includes MRI and histological examination of an endometrial sample obtained by curettage with hysteroscopy or biopsy with a Cornier® pipelle. The assessment determines the tumour stage, histological type and grade for endometrioid (type I) adenocarcinomas [1]. The aim of the preoperative assessment is to classify patients into one of three risk groups (low, intermediate or high) for lymph node invasion and recurrence and to help guide surgical staging to determine whether pelvic and/or para-aortic lymphadenectomy should be performed [1–10]. Although no benefit for lymphadenectomy was seen in patients with early low-risk disease in two prospective randomised trials [11, 12], lymph node status is a strong prognostic factor for guiding the choice of adjuvant treatment [9, 13–18].

The preoperative evaluation of lymph node metastatic risk based on MRI is poor because used criteria are defective or incomplete [19–21]. For example, using MRI, myometrial involvement > 50 % is underestimated in 21 % and 32 % of endometrioid grade 1 and grade 2 adenocarcinoma, respectively [22]. In the same way, the grade is underestimated by preoperative biopsy or curettage in 20 to 25 % of preoperative endometrioid grade 1 adenocarcinoma [23]. Although this defective performance of each tool assessing preoperative risk, little data are available that evaluate combined MRI and preoperative biopsy to assess endometrial cancer risk for lymph node invasion and recurrence.

The aim of this study was to evaluate the combined performance of MRI and histological analysis in predicting the risk of lymph node invasion and recurrence. Used preoperatively, the combined assessment can identify which patients should undergo pelvic and/or para-aortic lymphadenectomy as per the existing guidelines [1].

## Methods

### Design

This is a retrospective study of all patients who underwent surgery for endometrial cancer between 1st January 2010 and 31st December 2013 at either Rennes University Hospital or Vannes Regional Hospital (by 5 general gynaecologic surgeons). The inclusion criterion was evidence of endometrial cancer of any type, grade or stage as confirmed by a preoperative endometrial sample or hysterectomy specimen. The exclusion criteria were lack of preoperative data concerning the type and grade (determined by endometrial sampling) or FIGO stage (determined by MRI). The research protocol was approved by the Institutional Review Board of the French College of Obstetrics and Gynaecology (CEROG 2014-GYN-020).

### Data collection

The following data were collected for all patients: patient characteristics (age, BMI, menopausal status, use of hormone replacement therapy for the menopause), surgical treatment (type of procedure, approach), adjuvant treatment, preoperative radiology and histology results, as well as final histology.

The preoperative MRI was performed following European Society of Urogenital Imaging guidelines [24]. The data required for FIGO 2009 staging (degree of myometrial, cervical, adnexal and serosal invasion, positive pelvic or para-aortic lymph nodes greater than 10 mm in size) were also collected. The data collected from the preoperative histology report were tumour type and grade for endometrioid tumours (type 1) and the method used: Cornier® pipelle (an outpatient procedure) or curettage with hysteroscopy (performed in the operating theatre). Following final analysis of the surgical specimen, the type, grade, presence of lymphovascular emboli and FIGO 2009 stage were recorded.

### Definition of risk levels for lymph node invasion

Type 1 tumours consisted of endometrioid adenocarcinomas and mixed tumours with a mucinous or villoglandular component in addition to the endometrioid component. For these tumours, histological grade was defined by the percentage of the undifferentiated component: grade 1 corresponded to an undifferentiated component of less than 5 %, grade 2 from 6 to 50 % and grade 3 more than 50 %. When nuclear atypia was marked, the grade was increased by 1 point [1].

Type 2 tumours were those with at least one serous, clear cell or carcinosarcoma component. Tumours that were completely undifferentiated or whose type could not be determined were classified as type 2.

In both the preoperative and postoperative periods, on the basis of their FIGO 2009 stage, type and grade the tumours were classified into recurrence risk groups as defined in the European Society for Medical Oncology (ESMO) guidelines [5, 6]. The three risks groups were determined as follows: low risk – stage IA, grade 1 or 2, histological type 1; intermediate risk – stage IA, grade 3 and stage IB, grade 1 or 2, histological type 1; high risk – stage IB, grade 3 and, by extension, stage ≥ II, histological type 1, as well as all type 2 tumours of any stage and tumours of any type or stage with lymphovascular emboli, as per French guidelines [1].

### Outcome measure

The primary endpoint was the risk group (low, intermediate or high). Secondary endpoints were FIGO stage, myometrial, cervical and lymph node involvement as well as tumour type and grade.

### Statistical analysis

The results of the preoperative assessment were compared with the final histological analysis of the surgical specimen. For type and grade assessment, the performance of the Cornier® pipelle and curettage with hysteroscopy was compared.

The sensitivity, specificity, PPV, NPV, LR+ and LR- of the preoperative assessment were calculated for each endpoint, together with 95 % confidence intervals (95 % CI). The percentage of underestimation or overestimation and accuracy rate were also calculated. The higher the LR+, the more useful the positive result of an examination proved to be. The examination was deemed useful when its LR+ was greater than 5. The closer the LR- was to 0, the more useful the negative result of the examination proved to be [1].

The *χ*^2^ test or Fisher’s exact test were used to compare categorical variables as appropriate. Results were considered significant when the *p* value was less than 0.05. All data were collected in an Excel database (Microsoft, Redmond, WA, USA) and analysed using R Core Team (2013) statistical software (R Foundation for Statistical Computing, Vienna, Austria; www.R-project.org).

## Results

The study identified 150 patients who had undergone surgery for endometrial cancer between 2010 and 2013: 99 (66.0 %) at Rennes University Hospital and 51 (34.0 %) at Vannes Regional Hospital.

Fifty-nine patients (39.3 %) were excluded for incomplete preoperative assessment data due to at least one of the following reasons: use of an imaging technique other than MRI (ultrasound, PET or CT scan) (*n* = 46), degree of myometrial invasion not specified on the MRI and images not available for review (*n* = 7), chance discovery of cancer in the surgical specimen (*n* = 9), type or grade not specified on the preoperative histology report and slides not available for review (*n* = 11).

**Fig. 1:**
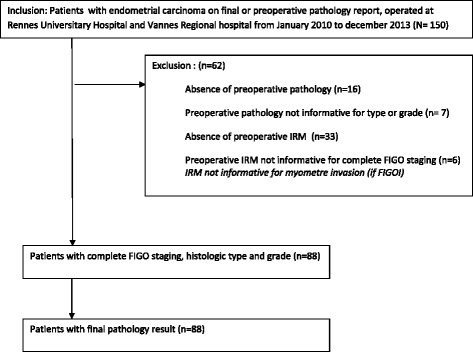
Flow chart

Ninety-one patients with endometrial cancer who had undergone a complete preoperative assessment (MRI to determine FIGO stage and histology to determine type and grade for type 1 tumours), were finally included in the study (Fig. 1).

The clinical and pathological characteristics of the patients are provided in Table 1.

**Table 1:** Characteristics of included patients (*n* = 91)

Variable	*n* /%
Age^a^ median and interval, in years	67 (38–90)
BMI^a^ median and interval in kg/m^2^	29.96 (19.23–53.83)
Menopause^a^	82 (90.11 %)
Hormonal treatment for the menopause^a^	26 (28.57 %)
Preoperative endometrial sample	
Curettage	44 (48.35 %)
Pipelle	47 (51.64 %)
Surgery performed^a^	
Total hysterectomy with BSO	87 (95.60 %)
Total hysterectomy	4 (4.40 %)
Surgical approach^a^	
Laparotomy	23 (25.27 %)
Laparoscopy	64 (70.33 %)
Vaginal	4 (4.40 %)
Lymphadenectomy^a^	
No	37 (40.66 %)
Pelvic	36 (39.56 %)
Pelvic + sentinel lymph node	3 (3.30 %)
Pelvic + para-aortic	13 (14.29 %)
Sentinel lymph node	2 (2.20 %)
Final histology results^a^	
FIGO stage (2009)^a^	
IA	45 (49.45 %)
IB	21 (23.08 %)
II	13 (14.29 %)
IIIA	2 (2.20 %)
IIIB	1 (1.10 %)
IIIIC	7 (7.69 %)
IV	2 (2.20 %)
Histological type^a^	
Type 1	78 (85.71 %)
Grade 1	46 (50.55 %)
Grade 2	20 (21.98 %)
Grade 3	12 (13.19 %)
Type 2	11 (12.09 %)
Absence of residual malignant cells	2 (2.20 %)
Lymphovascular emboli^a^	32 (35.16 %)
Risk of recurrence^a^	
Low	33 (36.26 %)
Intermediate	14 (15.38 %)
High	44 (48.35 %)

*BSO* bilateral salpingo-oophorectomy

^a^ There was no significant difference between the pipelle group and the curettage group

### Histological analysis

Table 2 compares the pre- and postoperative histological type.

**Table 2:** Histological type of endometrial cancers: comparison of results of the preoperative and postoperative analyses (*n* = 91)

Preoperative histological evaluation		*n*	Postoperative histological result		*n* (%)
Type 1	Endometrioid	80	Type1	Endometrioid	74 (92.5 %)
	Mucinous				
	Mixed endometrioid and mucinous			Mucinous	
				Mixed endometrioid and mucinous	
			Type 2	Mixed endometrioid and serous	4 (5.0 %)
			Other	Absence of residual tumour	1 (1.25 %)
				Atypical hyperplasia	1 (1.25 %)
Type 2	Serous	11	Type 1	Endometrioid	4 (36.4 %)
	Mixed endometrioid and serous or clear cells		Type 2	Serous	7 (63.6 %)
	Undifferentiated			Mixed endometrioid and serous or clear cells	

Of 80 patients with type 1 cancer (endometrioid) preoperatively, 4 (5.0 %) were reclassified as having type 2 cancer on final histological analysis. Conversely, of the 11 patients with type 2 cancer preoperatively, 4 (36.3 %) were reclassified as having type 1 cancer on final histological analysis. For the diagnosis of type 2 tumours, regardless of the technique used the endometrial sample had sensitivity of 63.6 % [95 % CI 35.4–84.8], specificity of 95 % [95 % CI 87.8–98.0], PPV of 63.6 % [95 % CI 35.4–84.8], NPV of 95 % [95 % CI of 87.8–98.0], LR+ of 12.7 [95 % CI 4.4–36.5] and LR- of 0.4 [95 % CI 0.1–0.8]. Regardless of the technique used, the endometrial sample correctly predicted the histological type in 89.0 % of cases, overestimated it in 6.6 % of cases and underestimated it in 4.4 % of cases (Table 3).

**Table 3:** Diagnosis of type 2 endometrial cancers by preoperative endometrial sample (*n* = 91)

	Cornier® Pipelle (*n* = 47)	Curettage with hysteroscopy (*n* = 44)	Both types of sampling (*n* = 91)
Sensitivity (%) [95 % CI]	60.0 [23.1–88.2]	66.7 [30.0–90.3]	63.6 [35.4–84.8]
Specificity (%) [95 % CI]	97.6 [87.7–99.6]	92.1 [79.2–97.3]	95.0 [87.8–98.0]
PPV (%) [95 % CI]	75.0 [30.1–95.4]	57.1 [25.0–84.2]	63.6 [35.4–84.8]
NPV (%) [95 % CI]	95.3 [84.5–98.7]	94.6 [82.3–98.5]	95.0 [87.8–98.0]
+LR [95 % CI]	25.2 [3.2–198.6]	8.4 [2.5–28.7]	12.7 [4.4–36.5]
-LR [95 % CI]	0.4 [0.14–1.2]	0.4 [0.1–1.1]	0.4 [0.1–0.8]
Overestimation (%)	2.1 *	11.4*	6.6
Accuracy rate (%)	93.6*	84.1*	89.0
Underestimation (%)	4.3*	4.5*	4.4

*PPV* positive predictive value, *NPV* negative predictive value, *+LR* positive likelihood ratio, −*LR* negative likelihood ratio

*There was no significant difference between the pipelle group and the curettage group (*p* = 0.259)

The endometrial sample was taken with a Cornier® pipelle in 47 patients (51.6 %) and by curettage in 44 patients (48.3 %). Diagnostic performance for type 2 tumours is provided in Table 3. No significant difference was observed between the two techniques for diagnosis of histological type.

The diagnostic performance of grading was assessed in the 74 patients with a tumour diagnosed preoperatively as type 1 and confirmed postoperatively. Regarding endometrioid tumours, for the diagnosis of grade 3 tumours the endometrial sample had a sensitivity of 30.0 % [95 % CI 10.8–60.3], specificity of 98.4 % [95 % CI 91.7–99.7], PPV of 75.0 [95 % CI 30.1–95.4], NPV of 90.0 % [95 % CI 80.8–95.1], LR+ of 19.2 [95 % CI 2.2–166.9] and LR- of 0.7 [95 % CI 0.4–1.1]. Endometrial sampling correctly predicted the grade for type 1 tumours in 89.2 % of cases, overestimated it in 1.3 % of cases, and underestimated it in 9.5 % of cases (Table 4).

**Table 4:** Diagnosis of grade 3 endometrial cancers, among endometrioid-type cancers, by preoperative endometrial sample (*n* = 74)

	Cornier® Pipelle (*n* = 41)	Curettage with hysteroscopy (*n* = 33)	Both types of sampling (*n* = 74)
Sensitivity (%) [95 % CI]	28.6 [8.2–64.1]	33.3 [6.1–79.2]	30.0 [10.8–60.3]
Specificity (%) [95 % CI]	97.1 [85.1–99.5]	100 [88.6–100]	98.4 [91.7–99.7]
PPV (%) [95 % CI]	66.7 [20.8–93.9]	100 [20.7–100]	75.0 [30.1–95.4]
NPV(%) [95 % CI]	86.8 [72.7–94.2]	93.7 [79.9–98.3]	90.0 [80.8–95.1]
+LR [95 % CI]	9.7 [1.0–92.9]	NA	19.2 [2.2–166.9]
-LR [95 % CI]	0.7 [0.5–1.2]	NA	0.7 [0.4–1.1]
Overestimation (%)	2.4*	0.0*	1.3
Accuracy rate (%)	85.4*	93.9*	89.2
Underestimation (%)	12.2*	6.1*	9.5

*PPV* positive predictive value, *NPV* negative predictive value, *+LR* positive likelihood ratio, −*LR* negative likelihood ratio, *NA* not applicable

*There was no significant difference between the pipelle group and the curettage group (*p* = 0.555)

The performance of the pipelle and curettage for diagnosing grade 3 tumours among endometrioid tumours is provided in Table 4. There was no significant difference between the two sampling techniques for establishing the grade.

Lymphovascular emboli were found in 32 out of 91 patients (35.3 %) on histological analysis of the hysterectomy specimen. Emboli were detected in 20 % of stage IA, 38 % of stage IB, 38 % of stage II, 80 % of stage III and 100 % of stage IV tumours regardless of type.

### MRI analysis

The performance of the preoperative MRI for diagnosing lymph node, cervical and myometrial invasion is reported in Table 5. The degree of myometrial invasion was underestimated in 11.4 % of cases, accurate in 81.8 % of cases and overestimated in 6.8 % of cases. Cervical invasion was underestimated in 10.9 % of cases, accurate in 89.0 % of cases and never overestimated. Fifty-two of the 91 patients (57.1 %) underwent pelvic lymphadenectomy with para-aortic lymphadenectomy in 13 cases (14.3 %). Among these 52 patients, lymph node status was underestimated in 3.8 % of cases, accurate in 78.8 % of cases and overestimated in 17.3 % of cases.

**Table 5:** Diagnosis of deepness invasion of endometrial cancers by preoperative MRI (*n* = 91)

	Cervical invasion (*n* = 91)	Myometrial invasion ≥50 % (*n* = 91)	Lymph node invasion (*n* = 52)
Sensitivity (%) [95 % CI]	23.1 [8.2–50.3]	73.7 [58.0–85.0]	71.4 [35.9–91.8]
Specificity (%) [95 % CI]	100 [95.3–100]	88.0 [76.2–94.4]	80.0 [66.2–89.1]
PPV (%) [95 % CI]	100 [43.8–100]	82.4 [66.5–91.7]	35.7 [16.3–61.2]
NPV (%) [95 % CI]	88.6 [80.3–93.7]	81.5 [69.2–89.6]	94.7 [82.7–98.5]
+LR [95 % CI]	NA	6.1 [2.8–13.3]	3.6 [1.7–7.6]
-LR [95 % CI]	NA	0.3 [0.2–0.5]	0.4 [0.1–1.6]
Overestimation (%)	0	6.8	17.3
Accuracy rate (%)	89.1	81.8	78.8
Underestimation (%)	10.9	11.4	3.8

*PPV* positive predictive value, *NPV* negative predictive value, *+LR* positive likelihood ratio, −*LR* negative likelihood ratio, *NA* not applicable

For the diagnosis of FIGO stages > IA, MRI had a sensitivity of 78.3 % [95 % CI 64.4–87.7], specificity of 88.9 % [95 % CI 76.5–95.2], PPV of 87.8 % [95 % CI 74.5–94.7], NPV of 80.0 % [95 % CI 67.0–88.8], LR+ of 7.0 [95 % CI 3.0–16.3] and LR- of 0.2 [95 % CI 0.1–0.4] (Table 6).

**Table 6:** Diagnosis of intermediate- and/or high-risk endometrial cancers by preoperative assessment (MRI and endometrial sample) (*n* = 91)

	Endometrial sample		MRI	Combination (MRI and histology)	
	Type 2	Grade 3	FIGO Stage > IA	High risk	Intermediate and high risk
Sensitivity (%) [95 % CI]	63.6 [35.4–84.8]	30.0 [10.8–60.3]	78.3 [64.4–87.7]	47.7 [33.8–62.1]	70.0 [58.0–80.8]
Specificity (%) [95 % CI]	95.0 [87.8–98.0]	98.4 [91.7–99.7]	88.9 [76.5–95.2]	93.6 [82.8–97.8]	81.8 [65.6–91.4]
PPV (%) [95 % CI]	63.6 [35.4–84.8]	75.0 [30.1–95.4]	87.8 [74.5–94.7]	87.5 [69.0–95.7]	87.2 [74.8–94.0]
NPV (%) [95 % CI]	95.0 [87.8–98.0]	90.0 [80.8–95.1]	80.0 [67.0–88.8]	65.7 [53.7–75.9]	61.4 [46.6–74.3]
+LR [95 % CI]	12.7 [4.4–36.5]	19.2 [2.2–166.9]	7.0 [3.0–16.3]	7.5 [2.4–23.3]	3.8 [1.8–8.2]
-LR [95 % CI]	0.4 [0.1–0.8]	0.7 [0.4–1.1]	0.2 [0.1–0.4]	0.6 [0.4–0.7]	0.3 [0.2–0.5]

*PPV* positive predictive value, *NPV* negative predictive value, *+LR* positive likelihood ratio, −*LR* negative likelihood ratio

For the diagnosis of FIGO stages > IB, MRI had a sensitivity of 60.0 % [95 % CI 40.7-76.6], specificity of 93.9 % [95 % CI 85.4–97.6], PPV of 78.9 % [95 % CI 56.7–91.5], NPV of 86.1 % [95 % CI 76.3–92.3], LR+ of 9.9 [95 % CI 3.6–26.9] and LR- of 0.4 [95 % CI 0.2–0.6].

**Fig. 2:**
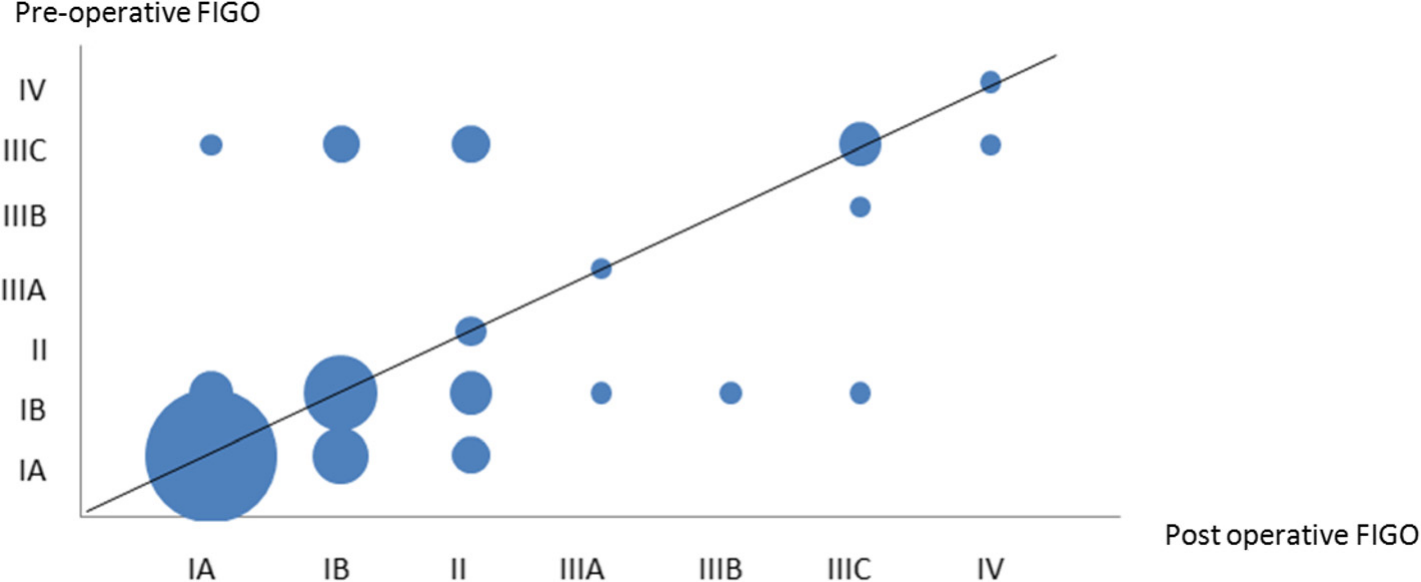
Pre and post operative assessment of FIGO stage. For the diagnosis of FIGO stages > IB, MRI had a sensitivity of 60.0 % [95 % CI 40.7–76.6], specificity of 93.9 % [95 % CI 85.4–97.6], Positive Predictive Value of 78.9 % [95 % CI 56.7–91.5], Negative Predictive Value of 86.1 % [95 % CI 76.3–92.3]

FIGO stages IA, IB, II, III and IV considered individually were underestimated in 20.9 % of cases, accurate in 65.9 % of cases and overestimated in 13.2 % of cases (Fig. 2).

### Performance of the preoperative assessment for determining risk

For diagnosing intermediate- or high-risk endometrial cancer, the preoperative combination of MRI and histology had a sensitivity of 70.0 % [95 % CI 58.0–80.8], specificity of 81.8 % [95 % CI 65.6–91.4], PPV of 87.2 % [95 % CI 74.8–94.0], NPV of 61.4 % [95 % CI 46.6–74.3], LR+ of 3.8 [95 % CI 1.8–8.2] and LR- of 0.3 [95 % CI 0.2–0.5] (Table 6).

Considered independently, the risk groups (low, intermediate and high) were underestimated in 31.9 % of cases, accurate in 60.4 % of cases and overestimated in 7.7 % of cases.

In practice, if we consider only the 23 patients who were in the low- or intermediate-risk group preoperatively but reclassified as high risk on final histology, the error was due to grade underestimation in 3 cases (14.2 %), underestimation of FIGO stage in 9 cases (42.8 %) and presence of emboli in 11 cases (47.8 %).

**Fig. 3:**
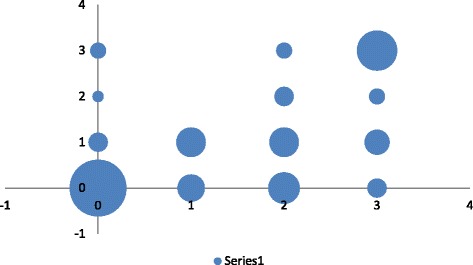
Pre and post operative assessment of risk. For diagnosing intermediate- or high-risk endometrial cancer, the preoperative combination of MRI and histology had a sensitivity of 70.0 % [95 % CI 58.0–80.8], specificity of 81.8 % [95 % CI 65.6–91.4], Predictive Positive Value of 87.2 % [95 % CI 74.8–94.0], Negative Predictive Value of 61.4 % [95 % CI 46.6–74.3]. 0 low risk, 1: intermediate risk, 2: high risk, 3 very high risk

Among the 37 patients with type 1, grade 1–2, stage IA adenocarcinoma on final analysis, 6 (16.2 %) were classified in the high-risk group owing to the presence of emboli. Similarly, among the 22 patients with type 1 grade 3 stage IA adenocarcinoma or grade 1–2 stage IB adenocarcinoma, 8 (36.4 %) were classified in the high-risk group due to the presence of emboli (Fig. 3).

### Surgical management according to risk group

In terms of surgical management, pelvic lymphadenectomy was not performed despite being considered in 6 patients classified in the intermediate- rather than low-risk group. Thirteen patients underwent pelvic lymphadenectomy only despite para-aortic lymphadenectomy also being recommended following final analysis. Ten patients did not undergo lymphadenectomy despite pelvic lymphadenectomy being recommended. Among these 23 patients, only 2 underwent a new procedure (one patient in the intermediate-risk group and one in the high-risk group) involving pelvic lymphadenectomy. No tumour invasion was observed in either case.

## Discussion

Our study showed that MRI underestimated the stage of endometrial cancer in 20 % of cases, and that endometrial sampling underestimated the type and grade in 4 % and 9 % of cases respectively. The combination of these two preoperative investigations does not compensate for any underestimation that may occur at an individual level. The risk group for lymph node invasion was underestimated in 31.9 % of patients, leading to surgical understaging with incomplete or no lymphadenectomy performed in 30.7 % of patients treated for endometrial cancer.

The limitations of our study are those inherent to its retrospective design: loss of data and selection bias. Interobserver variability in MRI and histology slide analysis can impair diagnostic performance. However our results are consistent with the literature [9, 25]. Several authors have studied the correlation between preoperative and final histology [9, 22, 25–32]. In terms of determining the tumour type, these studies report an accuracy rate of 74 % to 92 % [9, 28], with no significant difference between the different sampling techniques, a finding comparable to our own results. In terms of determining the grade, these studies report an accuracy rate of 44 % to 94 % [9, 22, 25, 28, 29], with no significant difference between the different sampling techniques either, a finding comparable to our own results [22, 25, 26, 28, 29]. Similarly, numerous authors have addressed the diagnostic performance of MRI for evaluating the degree of myometrial, cervical and nodal invasion [21, 33–37]. The results of our study are comparable to those in the literature [9, 25, 29, 37–42] which reports an accuracy rate of between 66 % and 90 % and a positive likelihood ratio >5 for evaluation of the degree of myometrial invasion [1]. Few studies have specifically evaluated the performance of MRI for FIGO staging, which is a composite criterion [9, 40, 42]. Raimond et al. [9] reported a modest performance with MRI for determining FIGO stage and a 30 % underestimation rate, consistent with the 20 % underestimation rate observed in our study. Their findings are similar to those of our study and confirm that the preoperative assessment performs better for more advanced and therefore higher risk stages. When predicting FIGO stage, overestimation by MRI is only 13 % in both our studies.

The concept of risk groups was introduced after 2000 [2, 5, 6, 13, 14, 43, 44] to estimate the risk of lymph node invasion and, over the longer term, recurrence, in early stage disease. The study by Bendifallah et al. [2] analysed the usefulness of different risk-group stratification models and concluded that none of the models was ideal for predicting recurrence and lymph node invasion. However risk stratification is still used to guide surgical management, and in particular the decision on whether or not to perform pelvic and/or para-aortic lymphadenectomy during total hysterectomy with bilateral salpingo-oophorectomy. Several stratification systems have been proposed, based on proven risk factors such as type, grade and stage, but also age and presence of lymphovascular emboli [2–7, 31, 45–48]. The latter criterion is an independent prognostic factor correlated with the risk of lymph node involvement and survival [31, 43, 46, 49, 50]. The 5-year overall survival rate for patients with emboli, of any stage, is 64 % as compared to 88 % for patients without emboli [5, 6]. Lymphovascular emboli should be considered in the risk prediction process to guide endometrial cancer management decisions. The inclusion of lymphovascular emboli in the risk prediction equation was responsible for half the cases of underestimation in our study — a finding consistent with the literature [51]. The underestimation is completely understandable given that emboli were found in 35 % of cases on final histological analysis of the surgical specimen and that they are undetected by preoperative histology. As shown by Hirschowitz et al., emboli are visible in the myometrium surrounding the tumour area but not in the tumour itself where tissue retraction is a source of confusion [31]. There has been no study on the possibility of detecting emboli preoperatively to date.

Our study and a previous one [2] show that the different models for stratification into risk groups for lymph node invasion are not useful for preoperative classification of patients. Lymphadenectomy is therefore not performed at the time of hysterectomy for 30 % of patients with endometrial cancer. The risk of overestimating the risk, i.e. performing an unnecessary lymphadenectomy, is low (estimated at 13 % in our series in agreement with the literature). Even if a preoperative assessment consisting of MRI and histology is not useful for estimating the risk of lymph node invasion, histological evaluation of lymph nodes is still considered desirable for patients with endometrial cancer who are classified as low-risk. To limit the morbidity associated with routine lymphadenectomy in patients with low-risk endometrial cancer, the sentinel lymph node technique may offer a useful alternative.

## Conclusion

The ESMO and of INCa guidelines propose therapeutic de-escalation for the management of endometrial cancer. Lymphadenectomy is not offered for low-risk endometrial cancer [1, 5, 6, 9]. However in patients with endometrial cancer classified as low-risk, risk stratification errors are observed on preoperative assessment, with underestimation occurring in at least 30 % of patients. Conversely, preoperative risk overestimation is low. In conclusion, in patients with endometrial cancer classified as low-risk as determined by a preoperative assessment consisting of MRI and endometrial sampling, histological examination of lymph nodes still proves useful. The sentinel lymph node technique could be used to limit the morbidity associated with routine lymphadenectomy and could be used routinely in patients with preoperative low risk endometrial cancer because of poor performance of preoperative assessment of combined MRI and endometrial sampling [21, 41, 52–60].
